# The relationship between the intraocular position of dexamethasone intravitreal implant and post-injection intraocular pressure elevation

**DOI:** 10.3389/fmed.2025.1582422

**Published:** 2025-09-08

**Authors:** Dingxi Liu, Yue Chen, Xiaonan Fu, Yongxia Zhao, Lili Ji, Yuanyuan Qiu, Sheng Li

**Affiliations:** Department of Ophthalmology, The Third People’s Hospital of Dalian, Dalian, China

**Keywords:** dexamethasone intravitreal implant, implantation position, intraocular pressure elevation, DEX-i, IOP

## Abstract

**Objective:**

To investigate the relationship between the implantation position of dexamethasone intravitreal implant (DEX-I) and post-injection intraocular pressure (IOP) elevation.

**Methods:**

This retrospective study included 324 patients (332 eyes) who received at least one DEX-I injection between June 2020 and June 2024, with a follow-up period of at least 3 months. Patient demographics, diagnoses, and DEX-I implantation positions were recorded. The correlation between implantation position and post-injection IOP elevation was analyzed. IOP elevation was defined as an IOP greater than 25 mmHg and/or an increase of 10 mmHg from baseline. DEX-I implantation positions were defined as follows: P1: implant located in the vitreous near the ciliary body, anterior to the ora serrata (with or without ciliary body contact); P2: implant located in the vitreous from the ora serrata to the pre-equatorial region; P3: implant located in the post-equatorial vitreous. The equator was defined by the vortex veins.

**Results:**

During the follow-up period, 68 eyes (20.48%) experienced IOP elevation. Compared to P2 and P3, the P1 implantation position was significantly associated with a higher incidence of IOP elevation (*p* < 0.001) and was positively correlated with early IOP elevation (within 15 days post-injection) (*r* = 0.761; *p* < 0.001).

**Conclusion:**

The P1 implantation is positively correlated with IOP elevation, particularly with early IOP elevation.

## 1 Introduction

Intravitreal injection has emerged as a significant milestone in ophthalmic treatment over the past two decades, widely used for various retinal diseases. Dexamethasone intravitreal implant (DEX-I, trade name: Ozurdex; Allergan, Inc., Irvine, CA, United States) serves as an important therapeutic agent for diabetic macular edema (DME), macular edema secondary to retinal vein occlusion (RVO), and non-infectious uveitis. Its efficacy and safety have been validated by numerous clinical trials (1–4). However, DEX-I has been reported to increase the risk of intraocular pressure (IOP) elevation in both randomized controlled trials and real-world studies. The primary mechanism of corticosteroid-induced IOP elevation is the increased resistance to aqueous outflow through the trabecular meshwork. The reported incidence of IOP elevation following DEX-I injection ranges from 20 to 70% (2, 3, 5), with variability attributed to several factors, including the position of the implant.

Previous studies have suggested that implants in contact with the ciliary body are associated with a higher rate of glaucoma surgery compared to those positioned in the posterior segment (6–8). At present, there are very few studies on the relationship between DEX-I different positions and IOP in the world, especially the data of Chinese patients is still in a blank stage. This study further explores the relationship between DEX-I implantation position and IOP through retrospective analysis of large samples, helping clinicians to understand the key of increased intraocular pressure that may be caused by DEX-I position, so as to optimize surgical operations and improve the safety of patient treatment.

## 2 Materials and methods

### 2.1 Study population

This retrospective study included 324 patients (332 eyes) who received at least one DEX-I injection between June 2020 and June 2024, with a follow-up period of at least 3 months.

Inclusion Criteria: Patients who received at least one DEX-I injection, with a minimum of 3 months of follow-up records, and where the DEX-I implant remained in the same position for the duration of 3 months.

Exclusion Criteria: Aphakic eyes; eyes with incomplete posterior capsules or zonular fibers; a history of glaucoma or a family history of glaucoma; other causes of abnormal IOP; abnormal trabecular meshwork morphology or function; a history of steroid-induced IOP elevation or a family history; concurrent use of other ocular steroids; vitrectomized eyes; and high myopia (defined as spherical equivalent refraction >−6.00 D).

### 2.2 Methods

Data collected included complete medical history, age, gender, diagnosis, baseline and post-injection visual acuity, intraocular pressure (IOP: non-contact tonometer TOPCON CT-800), results from anterior and posterior segment examinations, and Scanning Laser Ophthalmoscopy (SLO: Optos P200DTX) combined with indirect ophthalmoscopy (Windsor SL4 4AA and/or Ocular Maxlight 90D) to confirm the position of the DEX-I implant. Follow-up records documented visits at 15 days, 1 month, 2 months, and 3 months post-injection.

DEX-I implantation positions were defined as follows: Position 1 (P1): implant located in the vitreous near the ciliary body, anterior to the ora serrata (with or without ciliary body contact). Position 2 (P2): implant located in the vitreous from the ora serrata to the pre-equatorial region.

**Figure 1:**
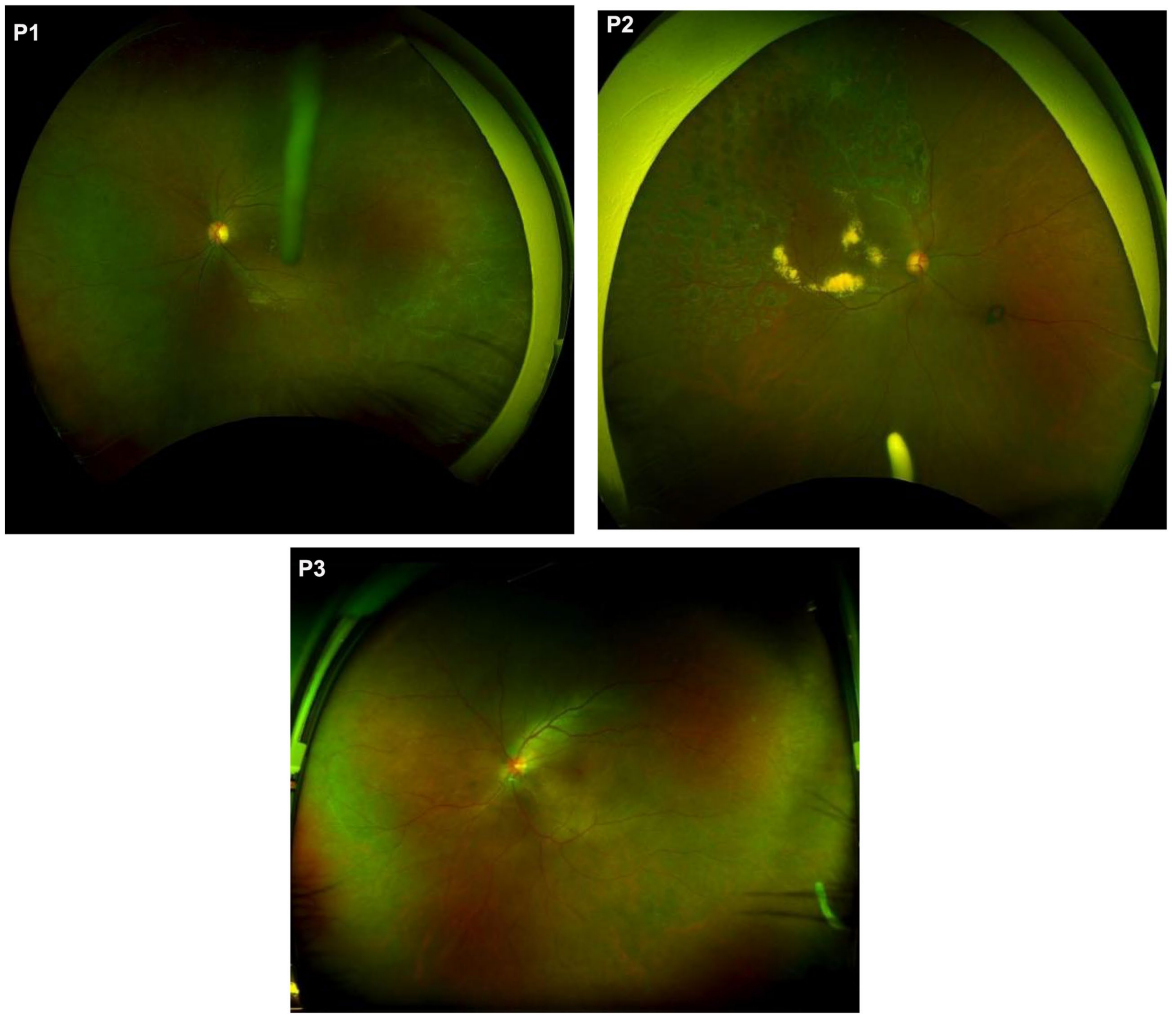
Scanning Laser Ophthalmoscopy (SLO) images showing different implantation positions. **(P1)** The white drug rod of DEX-I is situated in the anterior portion of the vitreous, adjacent to the ciliary body region. Due to its anterior location, the drug rod appears relatively large. **(P2)** The white drug rod ofDEX-I located in the vitreous from the ora serrata to the pre-equatorial region. **(P3)** The white drug rod of DEX-I is situated in the posterior vitreous of the inferotemporal region, appearing smaller due to its proximity to the retina.

Position 3 (P3): implant located in the post-equatorial vitreous. The equator is defined by the location of the vortex veins (Figure 1).

Post-treatment IOP elevation was defined as an IOP ≥ 25 mmHg and/or an increase of ≥ 10 mmHg from baseline (9). Extensive research indicates that an IOP ≥ 25 mmHg and/or an increase of ≥ 10 mmHg is deemed a critical risk factor for rapid progression, necessitating aggressive management (9–13). IOP elevation was graded as mild (increase < 6 mmHg), moderate (increase of 6–15 mmHg), or severe (increase > 15 mmHg) (14). Early IOP elevation was defined as IOP elevation occurring within 15 days post-injection.

### 2.3 Statistical analysis

Data were analyzed using SPSS version 25.0. Descriptive statistics included means, percentages, and variances. Parameter estimates were based on regression analysis, which provided model parameter estimates, standard errors, *p*-values, and 95% confidence intervals.

Univariate logistic regression analysis was employed to preliminarily screen factors potentially associated with IOP elevation, including gender, age, lens status, diagnosis, and implantation position. Subsequently, multivariate logistic regression analysis was conducted to identify independent risk factors while controlling for confounding variables, thereby providing a more accurate assessment of the impact of implantation position on IOP elevation. Correlation coefficients (r) were calculated to determine the strength of associations between variables. Fisher’s exact test was utilized to compare differences between diagnostic groups, with a *p*-value < 0.05 considered statistically significant.

## 3 Results

### 3.1 Demographics

A total of 332 eyes (324 patients) met the inclusion criteria, with a mean age of 65.32 ± 7.87 years. The primary diagnoses included DME (185 eyes, 55.72%), RVO (117 eyes, 35.24%), uveitis (20 eyes, 6.02%), and other conditions, such as Irvine-Gass syndrome (10 eyes, 3.01%). The distribution of implantation positions was as follows: P1 (67 eyes, 20.18%), P2 (148 eyes, 44.58%), and P3 (117 eyes, 35.24%) (Table 1).

**Table 1:** Baseline characteristics.

Baseline	DEX-I 0.7 mg (*n* = 332)
Age (years)	
Average age	65.32 ± 7.87
Gender	
Male	191(57.53%)
Female	141(42.47%)
Diabetes	264(79.52%)
Hypertension	251(75.6%)
Baseline IOP (mmHg)	14.26 ± 2.85
Clinical diagnosis	
DME	185(55.72%)
RVO	117(35.24%)
Uveitis	20(6.02%)
Others	10(3.01%)
Implantation position	
P1	67(20.18%)
P2	148(55.58%)
P3	117(35.24%)
Lens	
Pseudophakic	191(57.53%)
Phakic	141(42.47%)

### 3.2 IOP elevation

The mean baseline IOP was 14.26 ± 2.85 mmHg. During the follow-up period, 68 eyes (20.48%) experienced IOP elevation, with a mean age of 64.63 ± 7.93 years. Among these, 51 eyes (75%) exhibited mild elevation, 11 eyes (16.18%) demonstrated moderate elevation, and 6 eyes (8.82%) presented with severe elevation. The most common time for IOP elevation occurred 2 months post-injection (60.41%). Of the eyes with IOP elevation, 50 (73.53%) required one IOP-lowering medication, 13 (19.12%) required two medications, 4 (5.88%) required three medications, and 1 (1.47%) required three medications in addition to selective laser trabeculoplasty (SLT). No cases required surgical intervention. All five cases that required three medications or SLT were in the P1 group.

**Figure 2:**
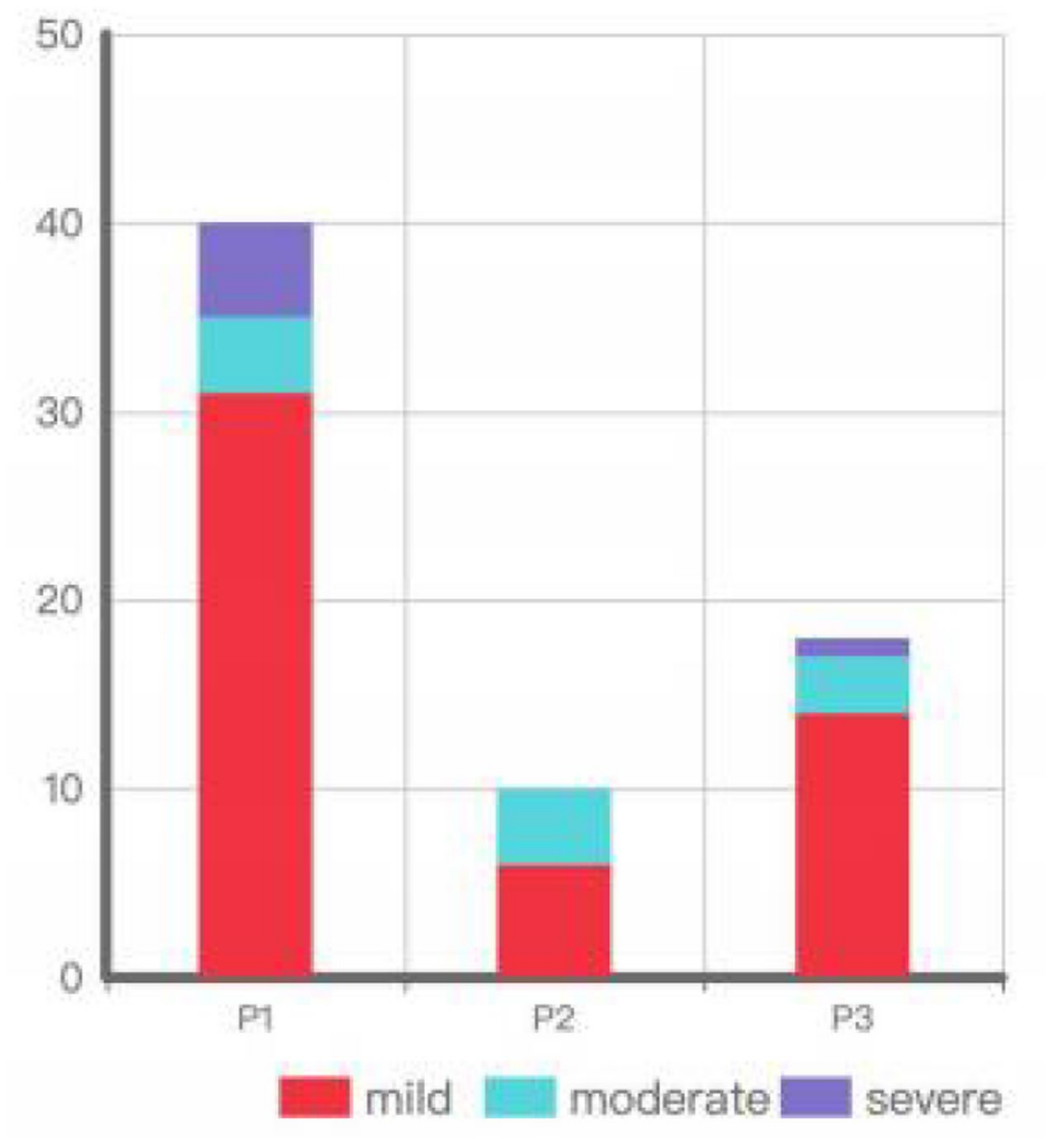
Degree of IOP elevation by implantation position.

Among the eyes with IOP elevation, 40 were in the P1 group (31 mild[77.5%], 4 moderate[10%], 5 severe[12.5%]), 10 in the P2 group (6 mild[60%], 4 moderate[40%]), and 18 in the P3 group (14 mild[77.78%], 3 moderate[16.67%], 1 severe[5.56%]) (Figure 2). Chi-square analysis showed no significant difference in the degree of IOP elevation among the different implantation positions (*p* = 0.495) (Table 2).

**Table 2:** Baseline characteristics by implantation position.

Characteristics	P1 (*n* = 67)	P2 (*n* = 148)	P3 (*n* = 117)	*P*
Average age (years)	63.76 ± 8.42	65.53 ± 7.95	65.94 ± 7.38	0.285
Baseline IOP (mmHg)	15.75 ± 3.36	13.97 ± 2.62	13.78 ± 2.53	<0.001
Gender				
Male (%)	35 (52.24)	60 (40.54)	46 (39.32)	
Female (%)	32 (47.76)	88 (59.46)	71 (60.68)	0.123
Diabetes (%)	45 (67.16)	143 (96.62)	76 (64.96)	<0.001
Hypertension (%)	51 (76.12)	108(72.97)	92 (78.63)	0.564
Clinical diagnosis				<0.001
DME	32 (47.76)	130 (87.84)	23 (19.66)	
RVO	31 (46.27)	14 (9.46)	72 (61.54)	
Uveitis	4 (5.97)	4 (2.70)	12 (10.26)	
Others	0 (0.00)	0 (0.00)	10 (8.55)	
Lens				0.32
Phakic	30 (44.78)	57 (38.51)	54 (46.15)	
Pseudophakic	37 (55.22)	91 (61.49)	63 (53.85)	

Statistical analysis revealed that male gender, the diagnosis of retinal vein occlusion (RVO), and P1 implantation were associated with IOP elevation (Table 3). Fisher’s exact test demonstrated that P1 implantation was significantly associated with IOP elevation compared to P2 and P3 (*p* < 0.001) (Table 4). Furthermore, early IOP elevation (within 15 days post-injection) was positively correlated with P1 implantation (*r* = 0.761; *p* < 0.001) (Table 5).

**Table 3:** Correlation of different variables with IOP elevation.

Variable	Correlation	*P*
Gender (male)	0.213**	<0.001
Uveit is	−0.003	0.956
RVO	0.235**	<0.001
DME	−0.209**	<0.001
P1	0.489**	<0.001
P2	−0.290**	<0.001
P3	−0.128	0.20

**Indicates significant correlation.

**Table 4:** Univariate and multivariate analysis of factors associated with IOP elevation.

Characteristics	Baseline characteristic		Univariate analysis		Multivariate analysis	
	OR	95% CI	*P*	Adjusted OR	95% CI	*P*
Age						
>60 years	Ref					
< 60 years	0.67	0.38 ~ 1.19	0.173	0.82	0.41 ~ 1.63	0.571
Gender						
Female	Ref					
Male	2.91	1.68 ~ 5.06	<0.001	3.28	1.69 ~ 6.36	<0.001
Lens						
Phakic	Ref					
Pseudophakic	0.68	0.4 ~ 1.16	0.16	0.67	0.35 ~ 1.29	0.66
Clinical diagnosis						
DME	Ref					
RVO	3.35	1.89 ~ 5.97	<0.001	3.97	2.11 ~ 7.47	<0.001
Uveitis	1.68	0.52 ~ 5.44	0.389			
Others	0.75	0.09 ~ 6.15	0.785			
Implantation position						
P3	Ref					
P1	8.71	4.29 ~ 17.71	<0.001	7.46	3.96 ~ 14.05	<0.001
P2	0.47	0.21	1.05	0.066		

**Table 5:** Correlation of different positions with early IOP elevation.

Variable	Correlation coefficient	*P*
P1	0.761**	<0.001
P2	−0.193	0.115
P3	−0.525**	<0.001

**Indicates significant correlation.

## 4 Discussion

IOP elevation following DEX-I injection has been a major concern for clinicians. In this study, 68 eyes (20.48%) experienced IOP elevation during the follow-up period. The GENEVA study (13) reported a 15.4% incidence of IOP > 25 mmHg, peaking at 60 days post-injection, while the SAFODEX study (15) reported a 20% incidence of IOP elevation, consistent with our findings. Zarranz-Ventura et al. (16) (*n* = 82) and Mayer et al. (17) (*n* = 64) reported a higher incidence of IOP elevation (approximately 40%), possibly due to their smaller sample sizes, which could be more susceptible to extreme values or specific cases, potentially leading to an overestimation of IOP elevation rates. On the *post hoc* analyses of the global phase III clinical trials for DEX-I (2, 13), we observed that the incidence of IOP elevation during patient follow-up did not increase with the number of injections administered, suggesting that DEX-I does not exert a cumulative effect on IOP elevation, and the impact of each injection on IOP can be regarded as a relatively independent event. Our study, which includes a larger sample size and encompasses various retinal diseases, provides more representative data, offering a more accurate reflection of the true incidence of IOP elevation following DEX-I implantation.

The primary mechanism of DEX-I-induced IOP elevation involves alterations in the ultrastructure of the trabecular meshwork. Dexamethasone inhibits protease activity and cellular phagocytosis, upregulates glucocorticoid receptors in the trabecular meshwork, and induces structural changes in trabecular meshwork cells. These changes lead to increased extracellular matrix deposition and resistance to aqueous outflow (18–20). Previous studies have reported a higher incidence of glaucoma surgery in cases where steroids were implanted in the ciliary body region compared to the posterior segment (6–8). Aditya Sudhalkar et al. (21) also noted that DEX-I implantation in contact with the ciliary body region is more likely to result in IOP elevation. Our study found that P1 implantation (located in the vitreous near the ciliary body, anterior to the ora serrata, with or without ciliary body contact) was significantly associated with IOP elevation, particularly early IOP elevation, compared to P2 and P3. This association may be attributed to the higher concentration of steroids in the anterior segment and the specific area of the trabecular meshwork affected by the steroids. Although our study did not reveal a statistically significant difference in the degree of IOP elevation among the different implantation positions (*p* = 0.495), five of the six cases with severe IOP elevation were in the P1 group, suggesting that P1 implantation may increase the risk of severe IOP elevation. However, due to the limited sample size of cases with severe IOP elevation, the statistical power was insufficient to fully and accurately reflect the relationship between P1 implantation and severe IOP elevation.

In this study, male gender and the diagnosis of retinal vein occlusion (RVO) were associated with IOP elevation. RVO is a significant risk factor for open-angle glaucoma, and increasing evidence suggests a reciprocal relationship between the two conditions. Muhtaseb et al. (22) reported a notable incidence of open-angle glaucoma following RVO, potentially linked to factors such as optic disc characteristics, retinal nerve fiber layer thickness, and microvascular perfusion. Previous studies have identified type 1 diabetes as a risk factor for steroid-induced IOP elevation; however, due to the limited number of type 1 diabetes patients in our DME cohort (only 2 cases), we did not conduct a separate analysis of the relationship between diabetes type and IOP elevation.

This study has several limitations due to its retrospective design. Among the 148 cases of P2 implantation, 130 were DME. Most RVO implantation were distributed in P1 and P3, with very few in P2 implantation. To the best of our knowledge, no prior studies have reported an association between the positional distribution of DEX-I and specific ocular diseases. As this is a retrospective study, we cannot definitively determine whether the observed distribution is coincidental or statistically significant. The absence of a standardized treatment and follow-up protocol may have resulted in inconsistencies in data collection. Additionally, the limited documentation of IOP elevation timing prevented a comprehensive understanding of the dynamic processes involved in IOP changes, complicating the analysis of the relationship between implantation position and IOP fluctuations. Nevertheless, the large sample size strongly suggests that the position of DEX-I implantation is an independent risk factor for IOP elevation, with P1 implantation showing a positive correlation with IOP elevation, particularly in the early stages. These findings have significant implications for clinical practice. Ophthalmologists should take the patient’s ocular condition into account when administering DEX-I injections, and whenever possible, avoid P1 implantation. For patients who do receive P1 implantation, close monitoring of IOP—especially during the early post-injection period—is essential to promptly detect and manage any elevation in IOP, thereby preventing potential damage to visual function and enhancing treatment safety.

## Data Availability

The original contributions presented in the study are included in the article/supplementary material, further inquiries can be directed to the corresponding authors.
